# A new frontier in temporomandibular joint osteoarthritis treatment: Exosome-based therapeutic strategy

**DOI:** 10.3389/fbioe.2022.1074536

**Published:** 2022-11-25

**Authors:** Wenxiu Yuan, Yange Wu, Maotuan Huang, Xueman Zhou, Jiaqi Liu, Yating Yi, Jun Wang, Jin Liu

**Affiliations:** ^1^ Lab for Aging Research, National Clinical Research Center for Geriatrics, West China Hospital, Sichuan University, Chengdu, China; ^2^ State Key Laboratory of Oral Diseases, National Clinical Research Center for Oral Diseases, Department of Orthodontics, West China Hospital of Stomatology, Sichuan University, Chengdu, China; ^3^ Department of Hepatobiliary Surgery and Fujian Institute of Hepatobiliary Surgery, Fujian Medical University Union Hospital, Fujian Medical University, Fuzhou, China

**Keywords:** temporomandibular joint osteoarthritis, Exosome, mesenchymal stem cell, therapeutic strategy, optimization in bioengineering

## Abstract

Temporomandibular joint osteoarthritis (TMJOA) is a debilitating degenerative disease with high incidence, deteriorating quality of patient life. Currently, due to ambiguous etiology, the traditional clinical strategies of TMJOA emphasize on symptomatic treatments such as pain relief and inflammation alleviation, which are unable to halt or reverse the destruction of cartilage or subchondral bone. A number of studies have suggested the potential application prospect of mesenchymal stem cells (MSCs)-based therapy in TMJOA and other cartilage injury. Worthy of note, exosomes are increasingly being considered the principal efficacious agent of MSC secretions for TMJOA management. The extensive study of exosomes (derived from MSCs, synoviocytes, chondrocytes or adipose tissue et al.) on arthritis recently, has indicated exosomes and their specific miRNA components to be potential therapeutic agents for TMJOA. In this review, we aim to systematically summarize therapeutic properties and underlying mechanisms of MSCs and exosomes from different sources in TMJOA, also analyze and discuss the approaches to optimization, challenges, and prospects of exosome-based therapeutic strategy.

## Introduction

**FIGURE 1:**
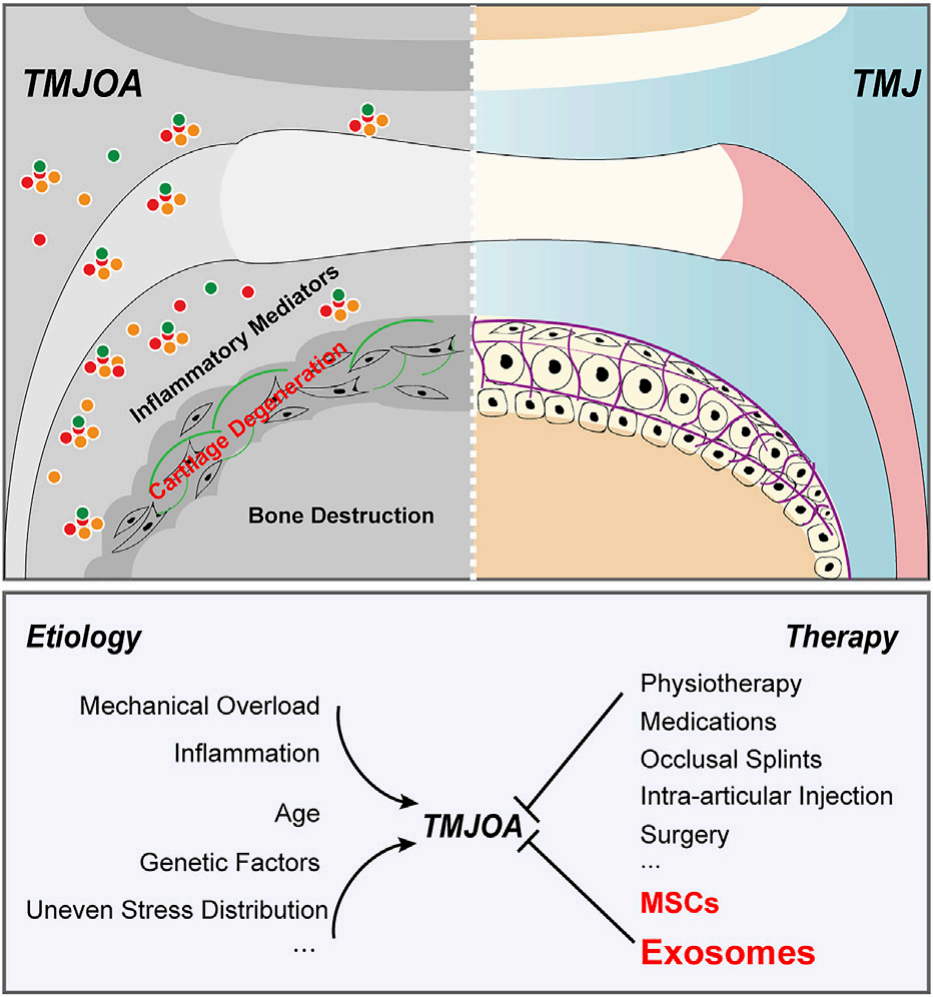
Pathogenesis and treatment strategies of TMJOA. TMJOA is a degenerative temporomandibular arthropathy characterized by progressive cartilage degeneration, abnormal subchondral bone remodeling and obvious synovitis. It is a pathological state affected by multiple factors. Traditional clinical strategies of TMJOA emphasize on symptomatic treatments and are unable to halt or reverse the destruction of cartilage or subchondral bone. MSCs and exosomes are highly promising for TMJOA alleviation.

Temporomandibular joint osteoarthritis (TMJOA) is a degenerative temporomandibular arthropathy characterized by progressive cartilage degeneration, abnormal subchondral bone remodeling and obvious synovitis (Scrivani et al., 2008; Toller, 1973). Due to the severe concomitant symptoms such as difficulties in chewing, acute or chronic pain, and even maxillofacial deformities, it severely deteriorates the quality of patient life and leads to the large resultant socioeconomic burden. Joint cartilage is composed of chondrocytes and extracellular matrix like collagen fibers, proteoglycans, and hyaluronic acid. Feature of avascular structure of cartilage is detrimental to the exchange of available signaling molecules, migration of progenitor cells, and adequate supply of nutrients and oxygen, resulting in the inability of damaged cartilage tissue to regenerate effectively (Chen et al., 2020). Because of the limited self-healing ability of cartilage, it has become one of the most difficult joint diseases to treat. Compared with other joints in the body, temporomandibular joint has its own characteristics (Macedo et al., 2017; David and Roberts, 2018). Besides, the layer of hyaline cartilage covering generalized joints mainly contained type II collagen, but the cartilage of TMJ is fibrocartilage, which is a kind of cartilage composed of both type I collagen and type II collagen. Because of the structural differences, there will be some differences in treatment strategies when the disease occurs. The cartilage of TMJ has better multidirectional bearing capacity and more dense fibers, which are not easy to degrade and are less affected by aging (Schwartz et al., 2015; Chandrasekaran et al., 2021). However, when it is damaged, the difficulty of restoring normal structure (Kuo et al., 2011). Numerous studies have confirmed that it is a pathological state affected by multiple factors (Figure 1). Excessive mechanical stress is a major factor leading to cartilage rupture in TMJ (Su et al., 2014; Huang et al., 2021; Ootake et al., 2021). Uneven stress distribution in TMJ caused by occlusal disorder was reported to induce the hyperactivity of osteoclasts in subchondral bone. Researchers have demonstrated that inflammation is one of the risk factors of TMJOA (Li et al., 2019a; Li et al., 2019b; Luo et al., 2019; Lei et al., 2022). Liu detected synovial fluid from TMJOA patients and found that the level of inflammatory cytokines was significantly increased.16 Moreover, genetic factors and age-related reduction of host-adaptive capacity are also vital in TMJOA (Xu et al., 2003; Yamaguchi et al., 2014). It is because of the specificality of TMJ structure and the ambiguity of etiological mechanism that the treatment of TMJOA has been set up a huge obstacle.

To date, treatment strategies for TMJAO are symptomatic and limited (Figure 1), only to reduce inflammation and relieve pain (Thie et al., 2001). Traditional clinical treatments can stop the progression of the disease to some extent, but they cannot actively restore degraded cartilage or damaged subchondral bone (Derwich et al., 2021; Liu Q. et al., 2022; Matheus et al., 2022). Novel radical therapies for osteoarthritis are urgently required. In recent years, cell-based disease treatment strategies have raised considerable concerns, especially mesenchymal stem cells (MSCs) -based therapies (Matheus et al., 2022). Abundant native MSCs are present in multiple niches in the joint, including subchondral bone, synovial fluid, and adipose tissue. In the last decade, increasing evidence has suggested that MSCs have great potential in the treatment of osteoarthritis. BMSCs have suggested promising therapeutic efficacy for TMJ cartilage repair (Ciocca et al., 2013). Although the role of MSCs in the field of disease treatment cannot be ignored, we still need to comprehensively understand its non-negligible bottlenecks as cell therapy strategies. The host exhibited immunological tolerance toward implanted MSCs and had a potential risk for malignancies, which might also pose a risk to immunological cells for controlling an inflammatory milieu (Lalu et al., 2012). Therefore, it is inevitable to find an alternative approach to solve the dilemma faced by MSC-based therapy. Numerous studies have summarized the bio-effect of MSCs is increasingly attributed to paracrine signaling to transfer its cargo to the body, among which exosomes are a vital carrier for message in many biological and pathological processes. Exosomes provide new perspectives for the development of cell-free and ready-to-use therapy for treatment of cartilage lesions and TMJOA.

Herein, the present review was aimed at discussing the therapeutic potential and corresponding mechanism of MSCs, the biological properties of exosomes derived from diversified cell sources, and advances in our knowledge of their emerging roles in managing TMJOA. We also discussed the detailed exosome-based tissue engineering strategies of TMJOA therapy in the hope of providing inspiration for future investigations. Particularly, we proposed novel perspectives for the development and implementation of exosomes as a cell-free regenerative medicine therapeutic strategy for cartilage repair in TMJOA and discussed future opportunities and challenges in this exciting field.

## Mechanisms of MSCs in the treatment of TMJOA

**FIGURE 2:**
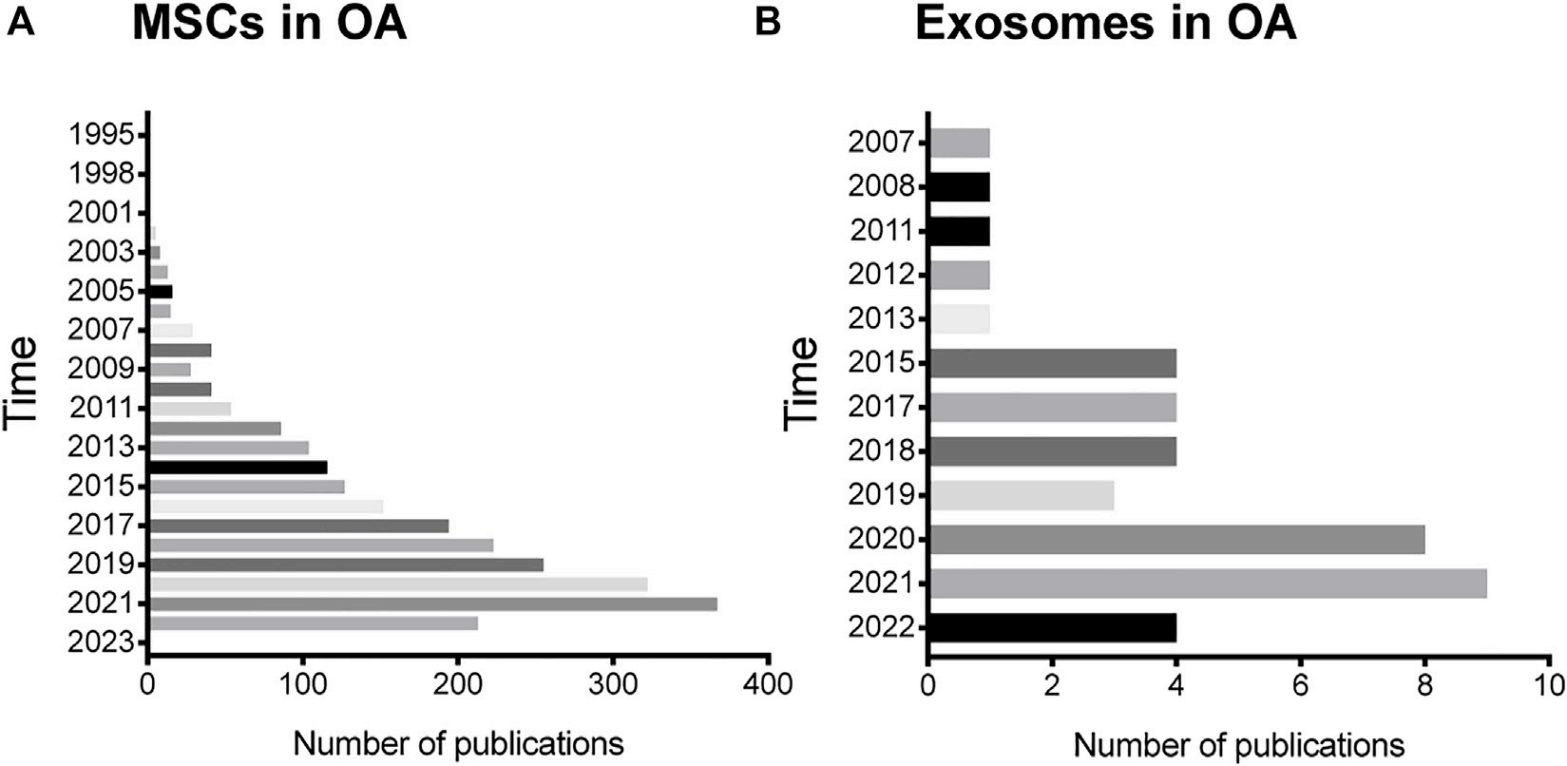
Status of MSCs and exosomes research in OA. **(A)** The annual number of publications related to MSCs research in OA in the past 27 years. **(B)** The annual number of publications related to exosomes research in OA in the past 15 years.

Since MSCs first discovery by Friedenstein (Friedenstein et al., 1982), they are commonly used in the treatment of various diseases, including TMJOA. We conducted a literature review and found that the application of MSCs in osteoarthritis was first reported in 1995, and more and more related research is being carried on, with over 89% of the published in the recent 10 years (Figure 2).

When TMJOA occurs, the dynamic balance between chondrocyte matrix anabolism and catabolism is disrupted, accelerating the progression of the disease (Weng et al., 2017). Zhang et al. (2017) reported BMSCs reversed the loss of cartilage matrix associated with osteoarthritis and enhanced scavenging activity of the degraded matrix in deep zone chondrocytes. Lu et al. (2015) also found that the implanted GFP-BMSCs differentiated into COL2-positive cells and relieved matrix degradation in TMJOA. It was indicated that human umbilical cord matrix-mesenchymal stem cells (hUCMSC) showed prominent cartilage protective effect and effective cartilage regeneration potential (Kim et al., 2019). Moreover, Maria revealed scaffolds loaded with dental pulp mesenchymal stem cells (DPSCs) effectively supported abundant fibrocartilaginous tissue formation. Besides, other MSCs, such as adipose-derived mesenchymal stem cells (ADMSCs) (Ahtiainen et al., 2013) and synovial fluid derived mesenchymal stem cells (SFDMSCs) (Koyama et al., 2011), have also been indicated to alleviate TMJOA by participating in cartilage matrix metabolism.

Uncoupled remodeling of subchondral bone is another pathological feature contributed to TMJOA (Jiao et al., 2011; Yang et al., 2014; Zheng et al., 2018; Ibrahim et al., 2019). Human exfoliated deciduous teeth stem cells (SHED) markedly improved surface smoothness and bone integrity of the destroyed condylar in TMJOA mice (Chen et al., 2013). Chen K investigated MSC-treated groups demonstrated pronounced micro-architectural changes of the subchondral bone (Tanaka et al., 2008). In addition, it was reported that the migration of BMSCs restored subchondral bone loss in mice with TMJOA (Lu et al., 2015).

Evidence has suggested that sustained inflammation is involved in the onset and progression of TMJOA (Liu W. et al., 2017). When stimulated, immune cells in inflammatory microenvironment release inflammatory factors to affect the matrix metabolism of chondrocytes to deteriorate TMJOA (Tanaka et al., 2008) and are also in close correlation with sensory neuron hyperexcitability to induce the pain of TMJOA (Magnano et al., 2007; Ou et al., 2021). Buul et al. found the decreased expression of IL-1β, MMP-1 and MMP-13 in synovial explants when cultured with MSCs conditioned medium (Van Buul et al., 2012). It was recently shown that BMSCs injection into the bilateral TMJ region significantly reversed high levels of TNF-α and IL-1β in TMJOA (Lu et al., 2015). This is further supported by Hyunjeong Kim’s study (Kim et al., 2019). The self-assembled peptide hydrogels accelerated tissue regeneration by anti-inflammatory modulation (Kim et al., 2016).

Inflammation and immunity go hand in hand (Bartholomew et al., 2002; Koliaraki et al., 2020; Lim et al., 2021; Pham et al., 2021). In the pathogenesis of inflammatory diseases, dysregulation of the host immuno-inflammatory response is one of the important predisposing factors (Theill et al., 2002; Hernández et al., 2011). Similarly, Monasterio proposed cytokines, CCLs and CCRs of the Th1/Th17/Th22 axis were involved in TMJOA pathogenesis (Monasterio et al., 2018). A large number of studies have shown that MSCs regulate innate and acquired immunity in the treatment of OA (Yu et al., 2016). Tang et al. (2021a) reported that hUCMSCs protected cartilage from injury by regulating the macrophages polarization and affecting the joint immune microenvironment, but notably, there was a stronger regulation ability of immune effector process in hUCMSCs-exosomes treatment group.

In recent years, although the efficacy of MSCs in treating TMJOA has been widely studied in animal studies and human clinical trials, in fact, the problems encountered in clinical application have been deeply troubling researchers (Table 1). Donor’s age affects the intrinsic activity and functionality of obtained cells (Kim et al., 2020). The lack of standardization for large-scale cell production results in inconsistent cell quality after expansion. Additionally, the senescence and dedifferentiation of cells during the expansion *in vitro* will also affect potential and increase the risk in the application (Siddappa et al., 2007). More cautiously, there is a potential of tumorigenicity (Le et al., 2012; Waterman et al., 2010). Moreover, the issue of cell storage is also a bottleneck of MSC-based strategy. Whether the biological activity of MSCs will be affected after repeated cryopreservation is a great question to be considered in future. It is noteworthy that there is a paradigm shift that, rather than direct differentiation to cells of the target tissue, the therapeutic efficacy of MSCs in tissue repair and regeneration is predominantly attributed to paracrine signaling, particularly exosomes (Mayourian et al., 2018; Li et al., 2019; Mori et al., 2019; Zhang et al., 2019; Zhou Q.-F. et al., 2020). Therefore, exosome-based therapeutic strategy of TMJOA may be a promising substitute for MSC-based therapy.

**TABLE 1:** Comparison of MSC-based and Exosome-based TMJOA therapies.

Treatment strategy	Acquisition	Transportation	Storage	Mass production	Delivery	Immunogenicity	Tumorigenicity	Treatment effect
MSC-based	Easy	Difficult	Difficult, cryopreservation affects cell viability	Time-consuming, cell senescence, change in biological characteristics	May happen cellular embolism	Certain degree	Certain degree	Good, clear
Exosome-based	Relatively easy, but the isolation is complicated	Easy	Easy, cryopreservation hardly affects affect exosome activity	Low yield, poor consistency	Not happen	Temporarily not found	Temporarily not found	Good, lack of clinical studies

MSC, mesenchymal stem cell; TMJOA, temporomandibular joint osteoarthritis.

## Characteristics of exosomes

It was not until 2006 that Ratajczak proposed for the first time that mRNA could be delivered by membrane-derived vesicles (MV) released from the surface of activated eucaryotic cells and exert positive effects on surrounding cells (Ratajczak et al., 2006). Exosome-mediated transfer of RNAs was suggested as a novel mechanism of genetic exchange between cells (Waterman et al., 2010), occurring within the microenvironment or at a distance by traffic of exosomes. Exosomes are the smallest in size ranging from 40 to 160 nm in diameter among the three main subcategories of extracellular vesicles (EVs). Exosomes of different cell origin carry their own various bioactive molecules, containing different types of proteins, DNAs, mRNAs, microRNAs, lipids, metabolites and so on. It is the diversity of contents that illustrates the diversity of exosome functions (Valadi et al., 2007). They are ubiquitously involved in the basic processes of innate and adaptive immunity and immune-mediated disease processes (Garikipati et al., 2018). It was shown that miR-21-5, as a lead cardioactive MSC-exosomal-microRNA, mediated effects on increasing engineered cardiac tissues contractility and was suggested as a specific molecular target for optimizing cardio-therapies (Mayourian et al., 2018). In recent years, increasing studies have been conducted on the application of exosomes in the treatment of neurological diseases (Budden et al., 2021; Xu et al., 2017; Rufino-Ramos et al., 2017). More attention should also be paid to bottlenecks in exosome treatment, including the limitation of increasing exosome production, the difficulty of analyzing the effective components of exosomes and the better improvement of the functions of the active component. Encouragingly, the problems of exosomes faced in the diseases therapy have been gradually handled *via* various biotechnology modifies. The composition and secretion of exosomes are affected by the environment and signals of donor cells, including hypoxia, heat, and pharmacological intervention (Pegtel et al., 2010; Fan et al., 2020). Therefore, changing the culture conditions of donor cells can meet the clinical needs of exosomes in treating diseases. Because exosomes are excellent carriers, the direct insertion of miRNA mimics or siRNAs into exosomes through electroporation (Ma et al., 2018) and electric pulses (Yang et al., 2020) has attracted the attention of many researchers. By modifying exosomes membrane through genetic manipulation strategy by biotechnology, exosomes can reach the target cells and tissues according to the predetermined route and play a more specific role (Kanki et al., 2011; Wang et al., 2018). The intersection between different cells exosomes and chondrocytes offered a new insight into the pathogenesis and treatment of degenerative joint diseases. Many studies have proposed that exosomes play an irreplaceable role in the treatment of TMJOA. In the following section, we summarized the current studies on the therapeutic effects of exosomes from various cells in TMJOA.

## Functional mechanisms and potential therapeutics of exosomes in TMJOA

**FIGURE 3:**
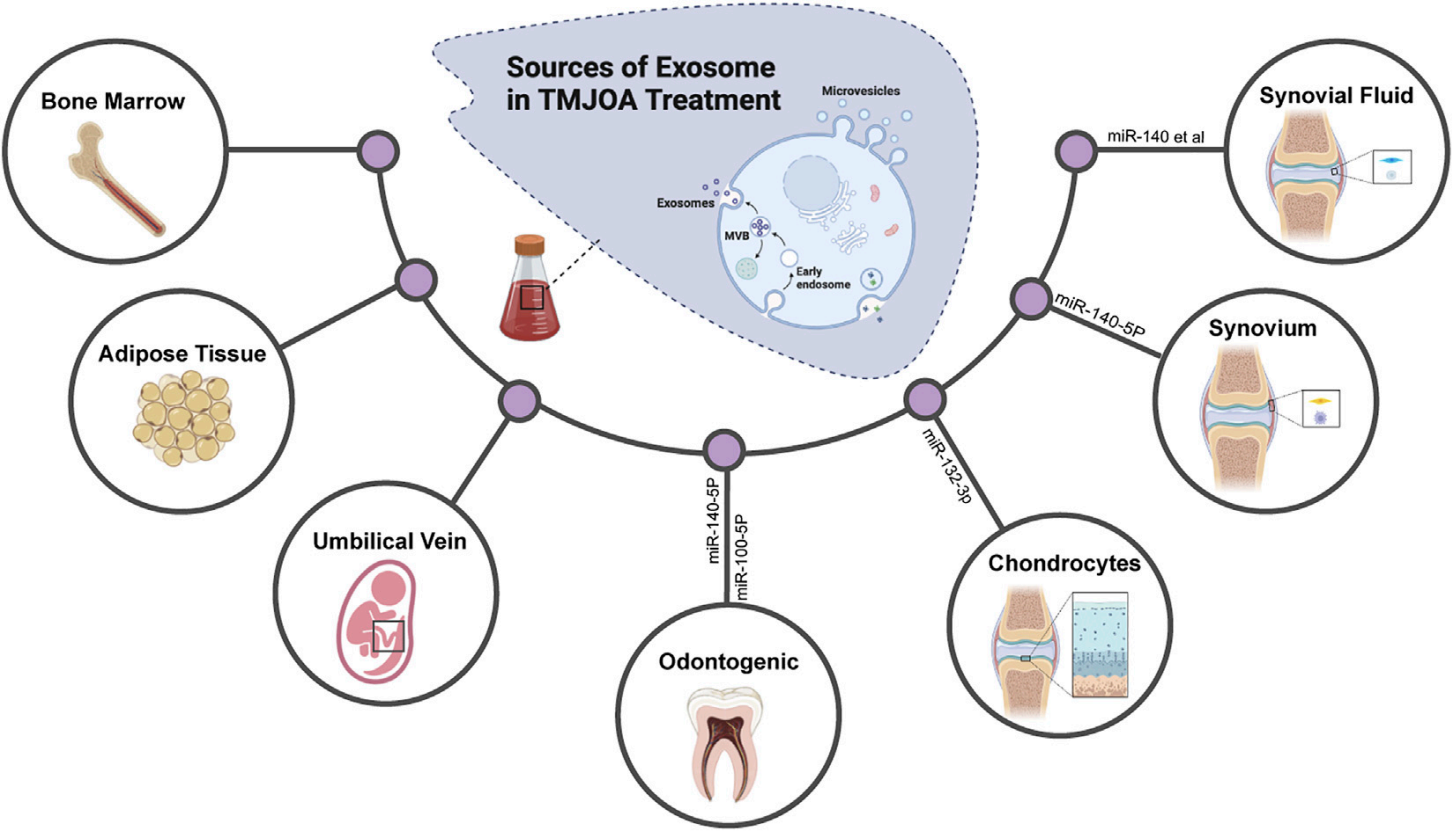
Exosomes derived from different tissues and cells are applied for TMJOA treatment. Diversified exosomes are obtained and applied in OA treatment to have a deeper understanding of the occurrence, development of OA, and to find more novel targets in molecular mechanisms of TMJOA treatment.

The different responses of recipient cells to exosomes are mainly due to the heterogeneity of exosomes, including their inconsistent expression of cell surface receptors and different contents. It means that exosomes from different cells have different effects on the same type of cell and the same exosome may also have inconsistent or even contradictory effects on different target cell types or target tissues66 (Table 2). More and more researchers attempt to obtain diversified exosomes and apply them in OA treatment to have a deeper understanding of the occurrence and development of OA and hope to find more novel targets in molecular mechanisms of TMJOA treatment (Figure 3). To date, an increasing amount of literature has indicated that exosomes from different sources (Figure 2), such as MSCs, chondrocytes, and synovial fluid in TMJ cavity, are reportedly important in the treatment of TMJOA. In recent years, researchers have focused on identifying effective constituents in exosomes, such as miRNA, for the treatment of TMJOA, with a view to obtain a more direct, effective, and targeted therapeutic strategy.

**TABLE 2:** Summary of Roles of Exosomes on Different Target cells in Osteoarthritis.

Target cell type	Sources of exosomes	Separation and extraction	Dose and delivery	Biological effects	Underlying mechanisms	Reference
Chondrocyte	BMSCs	Ultracentrifugation	20 μg, 40 μg *in vitro*; 40 μg/100 μl in vivo-IA	Proliferation; Migration; Matrix metabolism	Attenuate IL-1β-induced inhibition on proliferation and migration, downregulation of anabolic markers, and upregulation of catabolic markers	He et al. (2020)
	BMSCs	Ultracentrifugation	12.5 ng, 125 ng, 1.25 µg *in vitro*; 250 ng/5 µl in vivo-IA	Matrix metabolism; Apoptosis	Restore anabolic/catabolic equilibrium; Anti-apoptotic effect	Cosenza et al. (2017)
	SMMSCs	Ultracentrifugation	5 μg (10 × 10^11^ particles/ml) *in vitro*; 30 μ (10^11^ particles/ml) in vivo-IA	Catabolic metabolism	Promote proliferation and migration; Inhibited apoptosis	Wang et al. (2020)
	iPMSCs	Ultrafiltration	108particles/ml *in vitro*; 8 μl (1.0 × 10^10^particles/ml) in vivo-IA	Migration; Proliferation	Enhance the motility; Stimulate proliferation	Zhu et al. (2017)
	iPFPMSCs	ExoQuick-TC kit; Ultrafiltration	1, 5, or 10 × 10^8^ particles/ml *in vitro*; 10 μl (10^10^ particles/ml) in vivo-IA	Apoptosis; Migration; Metabolism; Autophagy	Inhibit apoptosis and promote anabolism; Enhance the level of autophagy *via* inhibition of mTOR pathway	Wu et al. (2019)
	Chondrocytes	Ultrafiltration	10 µg/ml, 20 µg/ml *in vitro*	Proliferation; Migration	Enhance proliferation and migration	Nikhil et al. (2022)
	Chondrocytes	Ultrafiltration	200 μg/ml *in vitro*; 200 μg in vivo-IA	Metabolism; Mitochondrial function	Restore chondrocyte metabolism; Eliminate mitochondrial dysfunction	Zheng et al. (2019)
	Chondrogenic progenitor cells	Ultracentrifugation	108 particles/ml *in vitro*; 8 μl (1.0 × 10^10^ particles/ml) in vivo-IA	Proliferation; Migration	Stimulate chondrocyte migration and proliferation *via* MiR-221-3p	Wang et al. (2020)
	Fibroblast-like synoviocytes	ExoQuick-TC Kit	Not reported	Proliferation; Migration; Matrix metabolism	Exosomal lncRNA H19 promotes cell viability and migration, and protects against ECM degradation by regulating miR-106b-5p and TIMP2 expression	Tan et al. (2020)
	Platelet-rich plasma	Ultrafiltration	200 µg/100 µl *in vitro*; 4 µg/2 µl in vivo-IA	Migration; Proliferation; Apoptosis; Degeneration	Promote proliferation, migration, and IL-1β-induced apoptosis and degeneration	Zhang et al. (2022b)
	Platelet-rich plasma; Hyperacute serum	Ultracentrifugation	1.42 × 10^9^ ± 2.12 × 10^6^ particles *in vitro*	Inflammation	Elicit chondroprotective gene expression; Inhibit inflammation by reducing IL-6 secretion	Otahal et al. (2020)
	IL-1β-treated chondrocytes	Ultracentrifugation	10 µg *in vitro*	Catabolic metabolism	Stimulate catabolic events	Liu et al. (2020)
	OA sclerotic subchondral bone osteoblast	Ultracentrifugation	10, 20, 50 µg/ml *in vitro*	Matrix metabolism; Cellular bioenergetics; Chondrocyte activity	Trigger the catabolic gene expression; Suppress the oxygen consumption rate *via* miR-210-5p	Wu et al. (2021)
	M2 phenotype macrophages	CM	Not reported	Formation; Differentiation	Downregulate chondrogenic-specific genes; Upregulate differentiation-related genes *via* LncRNA MM2P-induced, exosome-mediated transfer of Sox9	Bai et al. (2020)
	miR-126-3p-overexpressing synovial fibroblasts	Ultracentrifugation	2 × 10^9^ particles/ml *in vitro*; 40 μl (500 μg/ml) in vivo-IA	Proliferation; Colony formation; Inflammation	Suppress chondrocyte inflammation and apoptosis	Zhou et al. (2021)
	ATF4-modified serum	Ultrafiltration	10 μg/ml *in vitro*; 200 μg in vivo-IA	Proliferation; Apoptosis; Autophagy; Inflammation	Promote Proliferation and autophagy; Inhibit apoptosis; Decrease MMP13 and inflammatory cytokines	Cosenza et al. (2017)
Macrophage	Chondrocytes	Ultrafiltration	200 μg/ml *in vitro*; 200 μg in vivo-IA	Immune reactivity	Increase M2 macrophage infiltration with a concomitant decrease in M1 macrophages	Zheng et al. (2019)
	Osteoarthritic chondrocytes	Ultrafiltration	200 μl, 10^8^ particles/l *in vitro*; 10^9^ paritcals in 5 µl in vivo-IA	Inflammation; Autophagy	Stimulate inflammasome activation; Increase the production of mature IL-1β *via* miR-449a-5p/ATG4B-mediated autophagy inhibition	Ni et al. (2019)
	Inflamed synovial fluid	ExoQuick-TC Kit	7.5 × 10^9^ particles/ml *in vitro*	Inflammation; Matrix metabolism; Immune regulatory properties	Produce IL-1β and IL-16; Stimulate the production of CCL20, CCL15, and CXCL1 chemokines; Release MMP12 and MMP7	Domenis et al. (2017)
Synovial fibroblast	Apoptotic and activated T cells and monocytes	Differential Centrifugation	5 × 10^3^ microparticles, 5 × 10^4^ microparticles, 5 × 10^5^ microparticles *in vitro*	Inflammation; Matrix metalloproteinases	Increase the synthesis of inflammatory mediators and MMPs consistent with activation of NF-κB	Distler et al. (2005)
	BMSCs	ExoQuick-TC Kit	2 µg *in vitro*; 250 ng/5 µl in vivo-IA	Proliferation; Apoptosis; Inflammation	Weaken proliferation; Enhance apoptosis of synovial fibroblasts treated with IL-1β	Jin et al. (2020)
	TGF-β1-modified MSCs	Extraction kit	100 μl (1 × 10^11^ particles/ml) in vivo-IA	Polarization	Promote M2 polarization *via* carrying miR-135b targeting MAPK6	Wang et al. (2021)
Mesenchymal stem cell	Chondrocytes	Ultracentrifugation	10 µg *in vitro*	Differentiation	Promote chondrogenic differentiation	Liu et al. (2020)
	BMSCs	Ultrafiltration	200 μg/500 μl in hydroge *in vitro*; 100 μg in vivo-IA	Migration; Proliferation; Differentiation; Recruitment	Promote proliferation, migration, and chondrogenic differentiation; Stimulate BMSC recruitment *via* the chemokine pathways	Zhang et al. (2017)
	Platelet-rich plasma	Ultrafiltration	200 µg/100 µl *in vitro*; 4 µg/2 µl in vivo-IA	Migration; Proliferation; Differentiation	Promote proliferation, migration, and chondrogenic differentiation	Zhang et al. (2022a)
	Tenocyte	Ultracentrifugation	0.016, 0.08, 0.2, 0.4 μg *in vitro*	Proliferation; Differentiation	Induce the tenogenic differentiation through TGF-β; Promote proliferation	Xu et al. (2019)
	IL-1β-treated chondrocytes	Ultracentrifugation	10 µg *in vitro*	Differentiation	Inhibit chondrogenic differentiation	Liu et al. (20117)
Endothelial cell	IL-1β-stimulated synovial fibroblasts	Ultracentrifugation; ExoQuick-TC Kit	15 ml of conditioned medium	Migration; Tube formation activity	Promote migration and tube formation activity	Kato et al

BMSCs, bone marrow mesenchymal stem cells; IA, intra articular; SMMSCs, synovial membrane mesenchymal stem cells; iPMSCs, induced pluripotent mesenchymal stem cells; iPFPMSCs, infrapatellar fat pad mesenchymal stem cells; OA, osteoarthritis; CM, condition media.

### MSCs-derived exosomes

BMSCs have been used the earliest to treat various diseases because of their outstanding biological characteristics (Li et al., 2012; Li et al., 2020; Dubus et al., 2022). Certainly, BMSCs-derived exosomes have also been demonstrated to be ideal agents for the treatment of osteoarthritis. He Lei investigated BMSCs-exosome stimulation obviously reversed the inhibition effect of IL-1β on the proliferation and migration of chondrocytes, significantly upregulated the expression of COL2A and downregulated MMP13 *in vitro* and vivo (Armiñán et al., 2010). Although the previous studies were less homogenous due to problems with dose, injection frequency and management timing, one of the therapeutic functionalities of MSC-derived exosome is anti-inflammatory efficacy in promoting functional recovery of matrix metabolism homeostasis. After evaluation of the influences on injections of embryonic stem cell-derived-exosomes in TMJ-OA induced by monosodium iodoacetate (MIA), the underlying molecular mechanisms of exosome-mediated matrix homeostasis in TMJ injury repair and cartilage regeneration were clearly elucidated (Zhang et al., 2019). The obstacle of S-GAG synthesis induced by IL-1β was broken by the exosome treatment. Also, consistent with previous experiments, MSCs exosomes reduced inflammation by suppressing NO and MMP13. Together, MSCs derivative exosomes inhibit cartilage degeneration and TMJOA-induced pain by alleviating inflammation in the early stage, and then promote matrix proliferation and expression as well as the recovery of subchondral bone structure, and finally achieve the repair and regeneration of the overall TMJ.

In addition to alleviating inflammatory events in TMJOA, MSC-exosomes could also inhibit apoptosis of chondrocytes and activation of the immunity (Cosenza et al., 2017; He et al., 2020; Wang Y. et al., 2021). A study by Zhu revealed the exosomes from induced pluripotent stem cells (iPMSCs) or synovial membrane derived MSCs (SMMSCs) accelerated proliferation and migration of chondrocytes (Tang et al., 2021b). Notably, there was a proliferation promotion of chondrocytes in the co-culture studies of chondrocytes and MSCs (Zhu et al., 2017). Zhang found CD163^+^ cells of the cartilage overlying synovium in exosome-treated defect increased but CD86^+^ cells decreased, indicating higher M2 macrophages infiltration with a decline in M1 macrophages (Wu et al., 2011). Inflammatory cytokines, such as IL-1β, displayed a concomitant decrease like M1 macrophages. The senescence of chondrocytes appears generally during the progression of TMJOA (Clérigues et al., 2012; Zhang et al., 2018; Varela-Eirín et al., 2022). A wide range of evidence has shown that exosomes from ADMSCs declined the presence of phosphorylated histone H2AX, relieved DNA damage, restored the mitochondrial membrane changes and oxidative stress, and inhibited OA osteoblast senescence (Duarte, 2015). Additionally, numerous studies have indicated that there is a close relationship between autophagy and cartilage biology and the pathology of TMJOA (Almonte-Becerril et al., 2010; Lotz et al., 2011; Jeon and Im, 2017; Tofiño-Vian et al., 2017). It was demonstrated that infrapatellar fat pad (IPFP) MSCs-derived exosomes inhibited apoptosis and balanced the anabolic and catabolic processes of chondrocytes to prevent cartilage from damage by exosomal-miR100-5p-mediatied inhibition of mTOR-autophagy pathway (Ribeiro et al., 2016). The TMJ cavity is a highly complicated environment involving a variety of different cells. Undoubtedly, exosomes secreted by these cells play an important role in the regulation of microenvironment homeostasis.

### Chondrocytes-derived exosomes

The chondrocyte is the only cell type of cartilage and is critical in the maintenance of cartilage homeostasis. The effect of primary chondrocytes-derived exosomes on TMJOA has been confirmed (Ni et al., 2019; Wu et al., 2019; Zheng et al., 2019; Liu S.-S. et al., 2022; Nikhil and Kumar, 2022). Liu noticed exosome-like structures in abnormal calcified cartilage together with the decrease of matrix Gla protein and the increase of tissue-nonspecific alkaline phosphatase, CD63 and pyrophosphatase/phosphodiesterase-1 in TMJOA (Ni et al., 2019). After local injection of the exosome inhibitor, the process of calcification was inhibited. They speculated it was a new way in preventing and treating TMJOA to inhibit degenerative chondrocyte-derived exosomes. Chondrocytes-derived exosomes positively affected proliferation of chondrocytes and exhibited significant wound closure promotion due to roles in intercell communication. During the repair of cartilage injury, bilayered cryoge and chondrocytes-derived exosomes had a synergistic effect (Liu Q. et al., 2022). Furthermore, Zheng investigated the proteomics of primary chondrocyte exosomes and found the 2409 proteins of exosomes were involved in mitochondrial damage or dysfunction and immune system process. They attributed the protective effects of chondrocyte-exosomes on osteoarthritis to mitochondrial dysfunction elimination and M1-proinflammatory macrophages infiltration decrease with a concomitant M2- anti-inflammatory macrophage increase in cartilage (Nikhil and Kumar, 2022). It could be inferred from Liu’ results that the exosomes released by articular chondrocytes inhibited catabolism and increased mRNA levels of ACAN and COL2A as a facilitator of cell communication (Zheng et al., 2019). Chondrogenic progenitor cells (CPCs) have MSC characteristics with strong potential of cartilage differentiation and self-renewal ability (Liu et al., 2020). EVs secreted by CPCs from MRL/MpJ superhealer mice (MRL-EVs) had shown superior therapeutic capability in attenuating OA compared with control mice-EVs. MRL-EVs played a vital role in stimulating the proliferation and migration of chondrocytes (Koelling et al., 2009). After miRNA-seq analysis of exosomes, AMPK signaling, regulation of autophagy, and insulin signaling were observed to be associated with differentially expressed miRNAs and miRNA 221-3p was highly enriched in MRL-EVs.

### Synoviocytes-derived exosomes

Synovial inflammation is observed on magnetic resonance imaging of OA affected joints (Wang et al., 2020). There is increasing evidence that synovial inflammation is positively correlated with TMJOA severity (Roemer et al., 2010), and persistent low-grade synovial inflammation exacerbates cartilage damage (Raghu et al., 2017). Kato analyzed effects of synoviocytes-derived exosomes on chondrocytes and HUVECs (Kato et al., 2014). Compared with resting synoviocytes-derived exosomes, exosomes from IL-1β stimulated synoviocytes significantly promoted matrix catabolism and inhibited anabolism of chondrocytes. Migration and tube formation activity of HUVECs were improved. These findings indicated that SFCs exosomes represented a novel mechanism in the pathogenesis of osteoarthritis, which implied exosomes might be used as a therapeutic strategy for TMJOA. Dysregulated angiogenesis deteriorates the cartilage degradation, bone destruction and synovitis (Kato et al., 2014). Feng Yaping reported HMGB1 increased VEGF and HIF-1α in synovial fibroblasts of TMJOA and conditioned medium obtained from High-mobility group protein 1-treated TMJOA SFCs promoted the migration and tube formation of HUVECs (Chavakis et al., 2007). In addition, it was observed that synoviocytes-derived exosome-mediated cartilage repair was achieved by improvement in cell activity and migration ability as well as reduction of ECM degradation, of which synoviocytes-derived exosomal-lncRNA H19 suppressed the miR-106b-5p/TIMP2 axis (Feng et al., 2021). It was confirmed that the expression of miRNA-126-3p was sharply reduced in synovial fluid exosomes from OA patients. Exosomes derived from miR-126-3p-overexpressing synovial fibroblasts enhanced chondrocytes proliferation and suppressed chondrocytes apoptosis. What’s more, the exosomes significantly constrained the inflammation in chondrocytes by decreasing the IL-1β, IL-6, and TNF-α (Tan et al., 2020).

Synovial tissue maintains the basic composition and volume of synovial fluid. SFCs secrete synovial fluid, which in turn provides a low friction environment and nourishes surrounding tissues. Recently, many studies have focused on analyzing and comparing the synovial fluid derived exosomes differences between osteoarthritis patients and healthy people to find new molecular targets and related mechanisms for the treatment of osteoarthritis (Kolhe et al., 2017; Zhou et al., 2021). It has been proposed that miRNA contents differ between OA patients and healthy people. Moreover, there is a high gender-specific differential expression of miRNA in synovial fluid-derived exosomes in patients with OA (Zhou et al., 2021). Chondrocytes treated with OA-derived EVs had down-regulated expression of anabolic metabolism and elevated expression of catabolic metabolism and inflammatory molecules. Previous studies demonstrated that synovial fluid-derived exosomes of OA patients possessed the characterization of the proinflammatory profile to M1 macrophages. The exosomes upregulated the IL-1β expression and induced the release of chemokines and promoted the production of MMP7 and MMP12 (Kolhe et al., 2017).

### Subchondral osteocytes-derived exosomes

Subchondral bone supports the surface cartilage and bears the mechanical load. The crosstalk between the cartilage and subchondral bone is proceeding in an orderly manner, conducted in an exosome-dependent pattern (Domenis et al., 2017). Once the balance of the interaction is disrupted, cartilage breaks down and subchondral bone remodels abnormally, exacerbating the progression of OA (Wu et al., 2022). TMJ is one of the most flexible joints in the body and the subchondral bone of TMJ has an outstanding ability to withstand multidirectional forces. Sun discovered a new mode of osteoclast-osteoblast communication. MiR-214-enriched exosomes secreted by osteoclasts were specifically transferred into osteoblasts *via* ephrinA2/EphA2 axis and suppressed osteoblast function (Sanchez et al., 2005). Moreover, there was an obvious promotion of bone formation after osteoclast-targeted miR-214-3p inhibition (Sun et al., 2016).One In coculture, researchers found that chondrocytes endocytosed the osteoblast derived exosomes in osteoarthritis sclerotic subchondral bone and upregulated catabolic genes and downregulated chondrocyte-specific genes. Wu demonstrated miR-210-5p suppressed the oxygen consumption of chondrocytes and altered cellular bioenergetics, which could be a potential target for therapeutic intervention in OA (Li et al., 2016). It is suggested that targeting the exosomal-miRNAs-transfer of osteoclasts to chondrocytes is an entirely new treatment strategy. In early-stage osteoarthritis, an upregulation of exosomal-osteoclast-derived microRNAs drove the progression of the disease. However, blockage of osteoclast-originated exosomes retarded osteoarthritis progression, mechanistically, *via* increasing the resistance of chondrocyte to matrix degeneration, endothelial cell angiogenesis and axon sensory innervation (Xu et al., 2021). To explore the potential osteogenesis of the exosomes from osteoblasts, Ge isolated EVs from MC3T3 and presented osteogenesis-related proteins and pathways through the protein profile. Eukaryotic initiation factor 2 pathways played an important role in osteogenesis and represented a potential therapeutic avenue to tackle OA (Liu et al., 2020). Mineralizing osteoblasts-derived exosomes significantly promoted osteogenesis and influenced miRNA profiles in bone marrow stromal cells, which activated the WNT pathway by increasing β-catenin and dampening Axin1 (Ge et al., 2015).

### Adipose tissue-derived exosomes

Adipose tissue-derived exosomes are applicated in the treatment of various diseases (Xu et al., 2019; Zhou Q.-F. et al., 2020; Wei et al., 2020). Intra-articular adipose tissue functions to cushion the shock and acts as one of major sources of cytokines, active mediators as well as regenerative cells in repair. At present, research of adipose-derived exosomes for the treatment of OA mainly focused on adipose tissue-MSCs-derived exosomes (Koh et al., 2012; Ribeiro et al., 2016; Scheja et al., 2019). Sembronio et al. (2021) compared standard OA treatment with hyaluronic acid injections with the new TMJOA therapy of microfragmented adipose tissue injection using the Lipogems technology by a randomized clinical trial. Notably, pain reduction and mouth opening significantly improved in both groups. And the statistical analysis showed that the microfragmented adipose tissue injection group had a statistically significant advantage in the success rate of procedure compared with the hyaluronic acid injections group. Considering the number and secretion capacity of adipocytes, we speculate that their role in osteoarthritis is also critical because they may work as a graft in synovial, secretes exosomes and locally serve as a source of MSCs for a long time. However, a lot of investigations into adipocytes-derived exosomes are still needed to shed light on molecular mechanisms underlying pathogenesis.

### Other cell and tissue-derived exosomes

As early as in 2005, microparticles derived from T cells and monocytes were clearly reported to induce the synthesis of matrix metalloproteinases and inflammatory mediators in fibroblasts in a dose-dependent manner (Cui et al., 2016). These results provided evidence for vesicles derived immune cells promoting the destructive activity of SFs. It was reported that Sox9-containing-exosomes of monocytes stimulated with IL-4 or IL-13 upregulated COL2A and ACAN, promoted the differentiation of primary chondrocytes (Distler et al., 2005). Abnormality of the tendon was related to OA progression, which indicated that tendon repair might be another treatment for injury.40 In recent years, some studies have evaluated the role of tendon-derived exosomes in osteoarthritis. In the transwell system, paracrine factors released by tenocytes induced MSCs to the tenogenic differentiation in a TGF-β dependent manner and the inhibition of TGF-β pathway eliminated the effect (Bai et al., 2020).

Noticeably, since the 1970s, many studies have explored the mechanism of platelet rich plasma (PRP) in tissue repair (Toghraie et al., 2011). It contains a variety of cytokines and active substances, promoting tissue regeneration and healing (Xuan et al., 2020). So far, exosomes have been reported to exist in PRP and participate in related physiological and pathological processes (Dohan et al., 2008). Actually, PRP-derived exosomes have been applied to OA treatment *via* intra articular injection for years in preclinical studies. Zhang incorporated PRP-exosomes into thermosensitive hydrogel (Gel) and assessed its biological activity and the therapeutic effect on OA *in vivo* (Saumell-Esnaola et al., 2022). It promoted BMSCs proliferation, migration, and chondrogenic differentiation, and inhibited chondrocytes apoptosis and hypertrophy to delay the progression of osteoarthritis. Meanwhile, PRP-derived exosomes inhibited the TNF-α release from chondrocytes and presented a potential in alleviating OA *via* WNT/β-catenin pathway (Zhang Y. et al., 2022). Alexander found that citrate-anticoagulated platelet-rich plasma-derived exosomes displayed a higher expression of SOX9 protein and a better inhibition effect on proinflammatory cytokine release compared to hyperacute serum-derived exosomes (Liu et al., 2019). Besides, amniotic fluid (AF) is easily to obtain for application in tissue repair and regeneration. Researchers elucidated (Raghu et al., 2017) commonly expressed exosomal-miRNA of AF-derived exosomes, revealed RNA target genes were associated with senescence, fibrosis, and OA pathways, and suggested it as a therapeutic potential strategy for the treatment of osteoarthritis (Otahal et al., 2020).

To date, exosomes derived from different cells and tissues have exhibited effects on the occurrence, development, prevention, and treatment of TMJOA *in vitro* and *in vivo*. On the one hand, exosomes play a decisive role in controlling cartilage matrix homeostasis by promoting chondrocyte proliferation and migration and inhibiting chondrocyte apoptosis, thus reversing the deterioration of TMJOA, and alleviating the symptoms of TMJOA. On the other hand, the exosome is trigger in promotion of MSCs migration *via* various chemotactic pathways and can stimulate chondrogenic differentiation, and repairs cartilage defects. Besides, it cannot be ignored that they also have the great potential in regulating bone homeostasis to better support cartilage. A large number of studies have shown that changes of exosomes in the state of inflammation, which suggests that exosome-based disease treatment strategies will be effective. In the exosome based TMJOA therapy, they reduce the production of inflammatory factors and inhibit the differentiation of proinflammatory M1 macrophages and increase the ratio of M2 macrophages. However, it is just the beginning and further research is urgently needed to explore a more in-depth mechanism and perfect treatment strategy in TMJOA.

## Optimization in exosome-based bioengineering strategies of TMJOA therapy

Owing to the uniqueness and complexity of the TMJ, it is a great challenge to achieve complete restoration of its anatomical, structural, and functional integrity. The optimization of exosome-based strategy is a necessary step for TMJOA treatment. Bioengineering is constantly developing and provides an optimized solution in regenerative medicine (Table 3). Helgeland made a systematic review to answer the question of whether scaffold based TMJ tissue regeneration have better outcomes in TMJOA treatment. The overall preclinical evidence indicated that biomaterial scaffolds combined with biological components enhanced the potential for cartilage regeneration in TMJOA (Bellio et al., 2020). Additionally, the optimization of bioengineering technology in exosome based TMJOA therapeutic strategy has aroused the hot interests of researchers. There might be several disadvantages with direct administration of exosome-containing suspension in cartilage regeneration, especially, the difficulty of local exosome retention. Liu developed a photoinduced hydrogel exosome scaffold for a better retention of cargo exosome (Helgeland et al., 2018). In the system, they demonstrated that it retained stem cell-derived exosomes and showed an excellent biocompatibility and cartilage-integration by positively regulating both chondrocytes and BMSCs *in vitro* and promoting cartilage matrix and cell deposition at cartilage injury site. Using a crosslinked network of chondroitin sulfate, alginate-dopamine, and regenerated silk fibroin, an injectable hydrogel with encapsulated exosomes was exploited in superficial cartilage regeneration. Exosomes released by the hydrogels recruited BMSCs into defects *via* the chemokine pathway (Liu X. et al., 2017). These findings revealed the hydrogel coated with exosomes as a promising approach for accelerating cartilage regeneration *in situ* and neo-cartilage extracellular matrix remodeling. Chen designed a 3D printed cartilage extracellular matrix-gelatin methacrylate-exosome delivery scaffold (ECM/GelMA/Ex scaffold) with radial channels and superior cell recruitment capacity. The scaffold not only enhanced cartilage regeneration but also facilitated recovery of subchondral bone. Furthermore, they also found that MSCs exosomes enhanced mitochondrial biogenesis and rescued the mitochondrial dysfunction in degenerated cartilage (Zhang et al., 2021). The controlled exosome release platform with histological biological scaffolds solves the problems of insufficient local exosome concentration and short half-life of exosomes after injection to a large extent, optimizing the exosome based TMJOA treatment strategy.

**TABLE 3:** Bioengineering materials combined with exosomes for repair and regeneration of cartilage.

Biological material	Composition	Source of exosomes	Retention and release efficiency of exosomes	Delivery	Mechanism	Reference
3D printed scaffold with radially oriented channels	Gelatin methacrylate; Decellularized cartilage ECM	BMSCs	Retention: >56% for 14 days	Implantation in site of defect	Increase chondrocyte migration; Simulation of M2 macrophage polarization; Enhancement of cartilage and subchondral bone regeneration	Chen et al. (2019)
Acellular cartilage ECM with vertically oriented structure	Porcine articular cartilage	Wharton’s jelly derived MSCs	Not reported	Implantation in site of defect; Articular injection of exosomes	Promote BMSC and chondrocyte proliferation, BMSC migration and macrophage polarization toward the M2 phenotype	Jiang et al. (2021)
Photoinduced imine crosslinking hydrogel glue	O-nitrobenzyl alcohol moieties modified hyaluronic acids, Gelatin	Induced pluripotent stem cell line	Retention: >90% for 14 days; Release: 1 × 10^10^ particles/ml/day	Full-thickness defect with an *in situ* formed EHG tissue patch	Promote the migration and proliferation of chondrocytes and hBMSCs; Penetrate into the subchondral bone and formed a seamless interface-cartilage integration ability	Liu et al. (2017a)
Mussel-inspired hydrogel	Alginate-dopamine; Chondroitin sulfate; Regenerated silk fibroin	BMSCs	Release: 87.51% ± 3.71% for 14 days	Injection in site of defect	Promote the recruitment, proliferation and differentiation of BMSCs	Zhang et al. (2021)

The dense matrix biological barrier of cartilage makes chondrocyte-targeted drug delivery difficult. Exosomes enter the cell mainly *via* endocytosis, direct membrane fusion, or pinocytosis, 66 and the released contents could exert biological effects. It indicates exosomes have a great potential as a vehicle for drug delivery. Hence, it is an innovation and optimization in TMJOA treatment to modify surface structures for improving the efficiency of exosomes entering cells and to modulate encapsulated contents for strengthening therapeutic effects by genetic engineering technology or direct physicochemical loading. A study demonstrated that exosomes gained MSC targeting capability after fusing exosomal membrane protein Lamp 2b with MSC-binding peptide E7 (Chen et al., 2019). SFMSCs with E7-exosomes entered the middle zone of the cartilage more easily. Additionally, BMSCs-derived exosomes loading with KGN (a small molecule that can induce MSCs differentiation to chondrocytes) by electroporation efficiently increased COL2 and ACAN and induced higher cartilage differentiation of SFMSCs. *In vivo*, it showed best cartilage repair. ATF4-overexpressing exosomes were developed by introducing the mRNA of ATF4 into exosomes *via* electroporation. It showed these exosomes alleviated inflammation and cartilage degeneration in osteoarthritis mice by promoting chondrocytes autophagy and inhibiting apoptosis (Xu et al., 2021). To achieve a more predictable and desirable clinical response, specific therapeutic miRNA enrichment could be performed through the overexpressing genetic technique. It was suggested that miR-140-5p-overexpressing hSDMSCs derived exosomes enhanced the proliferation and migration abilities of chondrocytes (Wang Z. et al., 2021). They observed the exosomes highly expressed miR-140-5p blocked this side-effect in ECM *via* targeting RalA to enhance SOX9 and ACAN. Exosomes derived from miR-92a-3p-overexpressing MSCs targeted WNT5A to elevate chondrogenesis in MSCs and suppress cartilage degradation in primary chondrocytes (Tao et al., 2017). Besides, hBMSC-derived overexpressing miR-26a-5p exosomes relieved OA and were served as a repressor to retard damage of SFs *via* PTGS2 downregulation *in vitro* (Mao et al., 2018). Generally, utilizing a specific exosomal-miRNA mainly involves these proposed mechanisms, the overexpression of miRNA in cells, the isolation of exosomes containing miRNA, then delivery to chondrocytes in inflammatory microenvironment or TMJOA animal models, and finally targeting a pathogenic gene *via* miRNA. Some studies have shown that the effect of hypoxia-preconditioned exosomes on cartilage repair are superior to that of normoxia-preconditioned exosomes, manifesting in the promotion of chondrocyte proliferation and migration and the inhibition of chondrocyte apoptosis (Jin et al., 2020). TGF-β1-stimulated BMSCs-derived exosomes highly expressed miR-135b and polarized synovial macrophages (SMs) into M2 type to alleviate cartilage destruction. M2 polarization of SMs was significantly reversed by increase of MAPK6 (Zhang B. et al., 2022). Pretreating exosomes with physical or chemical stimulation optimizes exosome-based therapeutic strategies.

## Future opportunities and challenges of exosome-based therapeutic strategy in TMJOA

Although exosomes were originally regarded as useless metabolic byproducts, it is well recognized that exosomes, as various carrier of signaling mediators, play a vital role in mediating cell-to-cell communication and in activating immunomodulatory activity. Certainly, numerous studies have shown exosomes are sufficient to treat degenerative diseases, including systemic OA and TMJOA. A correct view of the prospects and existing problems of exosome-based therapeutic strategy is the basis for further research.

**FIGURE 4:**
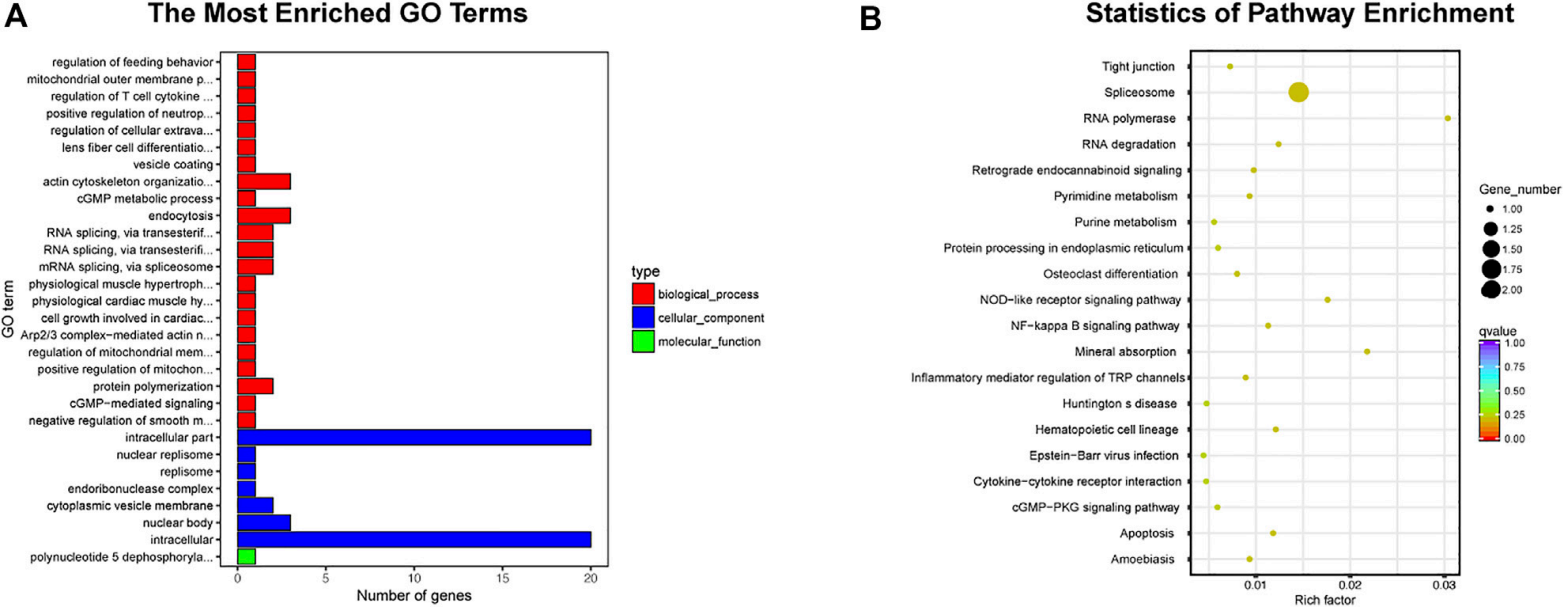
Enrichment analysis of exosome-derived miRNAs clearly related to TMJOA. **(A)** GO analysis, 30 enriched iterms are shown. **(B)** KEGG analysis, 20 pathways are shown.

Emerging as a trending research area, exosome-based therapeutic strategy in TMJOA has gained much interest because of its unique regulatory ability in TMJ inflammation as well as the low immunogenicity (Wang Y. et al., 2021). Some studies have reported that MSCs show a certain degree of immunogenicity in mediating disease treatment (Qi et al., 2016; Gregory et al., 2005). Compared with MSCs, exosomes have been reported not to express class II human leukocyte antigens and have lower immunogenicity when applicated *in vivo* (Krampera et al., 2006; Stagg et al., 2006; Sun et al., 2009; Geiger et al., 2015; Qi et al., 2016; Yaghoubi et al., 2019; Kang et al., 2020). TMJ is a complex system, and the immune privilege of exosomes maximally ensures it not to be cleared by immune cells when playing the therapeutic role in TMJOA. In addition, at present, few studies have reported the tumorigenic effect of exosomes *in vivo*, which might occur in the MSC-based therapy (Wislet-Gendebien et al., 2012; Funes et al., 2014; Li et al., 2022). Because cartilage is a dense biological barrier staggered by collagenous fiber, the transport property of exosomes is advantageous in TMJOA treatment. Being nano-sized and biocompatible, exosomes can be served as nanocarriers, easily reaching to the cartilage to fuse with chondrocytes. Certainly, the side effects associated with cell-based therapy, such as vascular embolism and pulmonary embolism (De Boeck et al., 2013; Jung et al., 2013), are also rare when exosomes are injected systematically. Furthermore, the less strict storage condition also gives exosomes greater possibilities for therapeutic application. Low temperature cold storage or repeated freezing and thawing does not influence exosome sizes and bioactivities, which shows higher clinical application value compared with cell-based therapies. It takes more time to resuscitate frozen cells to normal functional state, and the activity of resuscitated cells cannot be predicted (Mäkelä et al., 2015). Most importantly, many studies have shown that cell-cell interactions are mainly dependent on exosomes. MSCs-derived exosomes share the same or even more powerful biological effects than MSCs (Zhou X. et al., 2020), such as metabolic regulation of cartilage matrix, inhibition of inflammatory factors, relief of TMJOA pain, and homing of cells to the cartilage defect. Moreover, exosomes modified by genetic engineering have higher organotropism and cartilage-targeting capability. It has involved the comparison of exosomal differences in organs or systems between healthy and sick populations. High-throughput sequencing has confirmed that exosomes in the joint system differ between normal state and TMJOA, which indicates exosomes can be used as biomarkers for the early diagnosis of TMJOA. MiRNA is one of the important cargoes of exosomes. Through literature review, we noticed some exosome-derived miRNAs related with TMJOA treatment. RNAhybrid and miRanda databases were used to predict the target genes of the miRNAs, and Gene ontology (GO) and Kyoto Encyclopedia of Genes and Genomes (KEGG) enrichment analysis was performed for the identified target genes (Figure 4). It showed 22 biological processes enriched in the GO analysis, including endocytosis, actin cytoskeleton organization, protein polymerization, and other processes. And it displayed 20 signaling pathways obtained by the KEGG analysis, including osteoclast differentiation, inflammatory mediator regulation of TRP channels, apoptosis, and other pathways. The information of enrichment analysis suggested the mechanism and related targets of exosomes in the treatment of TMJOA, which will help us further study the pathogenesis of TMJOA and find out more effective TMJOA therapeutic strategy in the future.

Despite the excellent therapeutic effects of exosomes, there are many issues that need to be addressed. Unlike TMJOA cell-therapy, although results from preclinical studies have demonstrated the chondroprotection role of exosomes, explorations into the exosomes efficacy in treatment are still in the start-up stage. Currently, the research on TMJOA treatment mainly focused on small animals. There is almost no large animal studies or human clinical trials to evaluate exosome-based therapeutic strategy in TMJOA. Insufficient evidence from preclinical research and clinical trials significantly hindered the elucidation of mechanisms and the clinical translation applications. Therefore, future studies are recommended to bridge this knowledge gap and validate the safety and efficacy of exosomes therapy. Meanwhile, the difficulties encountered in the acquisition and the preparation of exosomes are inescapable. Various methods of exosomes separation *in vitro* have been developed, such as ultracentrifugation-based technique, size-based technique, and immuno-affinity action-based technique (Lőrincz et al., 2014). However, the most standardized and optimal operational procedure has not yet been established. The comparability between different studies is poor due to the differences of yield and purity of exosomes. Extracting homotypic exosomes with consistent contents is crucial in precisive therapy and in reduction of adverse effects caused by unintended unknown by-products (Ding et al., 2021). It is urgent to develop an optimal isolation procedure, which maximizes yield and purity of exosomes and minimizes changes of contents and sizes during extraction. In addition, due to the quick turnover of synovial fluid in TMJ cavity, more studies are needed to determine the effective dose and frequency of exosomes injection. Like cell-based therapy, exosome-based therapeutic strategy is also limited by rapid clearance *in vivo* and short effective period in direct injection. Therefore, it is particularly critical to optimize the therapeutic strategy of exosomes in TMJOA *via* tissue engineering approaches. Notably, the cartilage of TMJ is fibrocartilage, which is different from the hyaline cartilage of the most joints of the body. It is made up of various proportions of both cartilaginous tissue and fibrous and has a more complex tissue structure and tensile and compressive strength. Although there are many studies on the treatment of OA, it is still questionable whether these treatment measures are also effective for TMJOA. We should explore the effectiveness of these treatments on TMJOA in a more scientific and rigorous manner. ‘A one size fits all’ therapeutic scaffold may not achieve the best treatment effects in TMJOA (Embree et al., 2016; Jiang et al., 2021; Jiang et al., 2021; Fan et al., 2022). According to the characteristics of different fibrocartilage tissue types, layered scaffolds loaded with exosomes exhibit outstanding advantages in the formation of layered tissue structure in cartilage regeneration to simulate the normal fibrocartilage to the maximum extent. Though faced with challenges, exosome-based therapeutic strategy is promising in TMJOA and worthy of further investigations *in vivo* and *in vitro*.

## Conclusion

In this review, we summarized the roles of MSCs and exosomes in TMJOA, manifesting in the regulation of cartilage matrix metabolism, the balance of subchondral bone homeostasis, the relief of inflammation, and the effects of immune regulation. Currently, MSC-based therapy is facing many challenges, while exosome-based therapeutic strategy can be a promising novel alternative because of its advantages in cell-to-cell communication in TMJ system. Exosomes, as mini vesicles, deliver nucleic acids and proteins to target tissues or cells and exert therapeutic efficacy in TMJOA. The pathogenesis of TMJOA is complicated and multifactorial. The optimization of the existing exosome-based strategies, such as the combination of tissue engineering scaffolds or genetic modification, more effectively reduce the side effects involved in exosomes treatment and improve cartilage repair and regeneration. However, the translation from experimental research to clinical application of exosomes has been hindered due to insufficient evidence of preclinical and clinical trials. Further research is needed to identify effective constituents of TMJO-target exosomes and explore underlying mechanisms, to investigate therapeutic targets, to evaluate the safety of exosomes application, and finally to establish a consensus in the therapeutic potency of exosome-based therapeutic strategy.
